# Sleep mediates the association between homocysteine and oxidative status in mild cognitive impairment

**DOI:** 10.1038/s41598-017-08292-4

**Published:** 2017-08-10

**Authors:** Mayely P. Sanchez-Espinosa, Mercedes Atienza, Jose L. Cantero

**Affiliations:** Laboratory of Functional Neuroscience, Spanish Network of Excellence for Research on Neurodegenerative Diseases (CIBERNED), Pablo de Olavide University, Seville, Spain

## Abstract

Tremendous progress has been made over the last few years in understanding how sleep and amyloid-β (Aβ) cooperate to speed up the progression of Alzheimer’s disease (AD). However, it remains unknown whether sleep deficits also interact with other risk factors that exacerbate the pathological cascade of AD. Based on evidence showing that higher levels of homocysteine (HCY) and sleep loss increase oxidative damage, we here investigate whether the relationship between HCY and total antioxidant capacity (TAC) is mediated by changes in objective sleep in healthy older (HO, N = 21) and mild cognitive impairment (MCI, N = 21) subjects. Results revealed that reduced TAC levels in MCI was significantly correlated with increased HCY, shorter sleep duration, lower sleep efficiency, and reduced volume of temporal regions. However, only the HCY-TAC association showed diagnostic value, and this relationship was mediated by poorer sleep quality in MCI patients. We further showed that HCY-related cerebral volume loss in MCI depended on the serial relationship between poorer sleep quality and lower TAC levels. These findings provide novel insights into how impaired sleep may contribute to maintain the relationship between HCY and oxidative stress in prodromal AD, and offer empirical foundations to design therapeutic interventions aimed to weaken this link.

## Introduction

One of the hallmarks of aging is a pervasive accumulation of mitochondrial reactive oxygen species (ROS) followed by the decreased ability of cells to defend against and recover from oxidative insults^1^. Age-induced alteration of the cellular oxidative environment disrupts the homeostasis of the endoplasmic reticulum (ER), resulting in accumulation and aggregation of unfolded proteins, which in turn lead to a condition referred to as ER stress^2^. Given that age-related oxidative stress and impaired cellular stress adaptation increases the vulnerability of neurons to degeneration^3^, it is hardly surprising that mitochondrial dysfunction, impaired antioxidant enzymatic activity, and ROS overproduction are among the earliest events in Alzheimer’s disease (AD)^4^. Indeed, patients with mild cognitive impairment (MCI), considered the prodromal stage of AD, already show signatures of oxidative damage in both brain tissue^5^ and blood stream^6^.

Evidence suggests that other markers of MCI/AD, such as elevated levels of homocysteine (HCY)^7^ and impaired sleep^8^, may also exacerbate the oxidative stress response and AD pathology. Thus, increased levels of HCY have been shown to affect redox-signaling pathways in neuronal cells^9^ and instigate Aβ aggregation^10^, likely accounting for HCY-related brain atrophy patterns observed in MCI patients^11^. On the other hand, sleep loss has been associated with ROS-induced oxidative stress in middle-aged flies^12^, activation of a maladaptive ER stress response in old mice^13^, and Aβ aggregation in transgenic mouse models of amyloidosis^14^. However, it remains unknown whether the association between HCY levels and brain atrophy is influenced by the relationship between total antioxidant capacity and sleep quality. This study seeks to address this issue using overnight polysomnographic (PSG) recordings, high-resolution cerebral MRI scans, and peripheral blood levels of HCY and TAC in HO subjects and MCI patients.

The following hypotheses motivated our approach: 1) the association between HCY-TAC will be stronger in MCI than in HO. This assumption is based on the fact that a combined pattern of elevated HCY and decreased TAC was more specific to MCI/AD than increased HCY levels alone^15^, which is considered a non-specific risk factor for a variety of neurodegenerative conditions^16^; 2) the relationship between HCY-sleep will be present in both HO and MCI, as inferred from studies showing that elevated HCY levels are more frequent in subjects with a short sleep duration regardless of their clinical status^17^; 3) MCI patients will show a stronger relationship between sleep-TAC than HO subjects, as supported by evidence showing associations between shorter sleep durations and decreased TAC levels in different clinical populations compared with normal subjects^18, 19^; and 4) all these factors (i.e., HCY, TAC and sleep) will be related to each other in MCI because of their well-recognized effects on Aβ accumulation, which would account for HCY-sleep associated patterns of brain atrophy^11^.

## Materials and Methods

### Subjects

Twenty-one patients with amnestic MCI (6 females, mean age: 69.8 ± 6.5 yr) and 21 cognitively healthy older (HO) subjects (10 females, mean age: 67 ± 5.5 yr) were enrolled in the study. Participants were primarily recruited from senior citizen’s associations, memory screening programs, and hospital dementia services. Informed consent was obtained from all participants, and the Ethical Committee for Human Research at the Pablo de Olavide University approved all procedures in accordance with the Declaration of Helsinki.

All participants received a neurological examination. Subjects diagnosed with amnestic MCI showed an idiopathic amnestic disorder with absence of impairment in cognitive areas other than memory, and all of them met core clinical criteria for MCI due to AD with an intermediate level of certainty. Elderly depression was excluded based on the short version of the Geriatric Depression Scale (scores ≤ 5). Inclusion criteria for HO subjects were normal cognitive performance relative to appropriate reference values for age and education, Clinical Dementia Rating (CDR) global score of 0 (no dementia), and normal independent function, judged both clinically and by means of an interview for deterioration in daily living activities. None of the participants were taking cholinesterase inhibitors and/or sedative-hypnotic drugs at the time of recruiting or during the study, nor did they report sleep-disordered breathing, movement disorders during sleep, or unusual sleep schedules (e.g., due to nocturnal shift work), as confirmed by bed partners and/or caregivers.

### Measures of TAC and HCY

Overnight fasting serum samples were collected from all participants, stored at −80 °C, and thawed immediately before assay. To estimate serum TAC levels, we employed an improved oxygen radical absorbance capacity (ORAC) assay with fluorescein as the fluorescent probe^20^. This methodology provides a direct measure of hydrophilic chain-breaking antioxidant capacity against peroxyl radicals, and takes into account in the quantification both the inhibition time and inhibition percentage of free radical action. ORAC was determined in samples without proteins by diluting the serum with 0.5 M perchloric acid and centrifuging at 4 °C (14,000 rpm) for 8 minutes. The recovered supernatant was diluted 19-fold in 5 mM phosphate-buffered saline. Fluorescence was measured at 37 °C on a Victor X3 multilabel plate reader spectrophotometer (PerkinElmer, USA) at 485 nm and 535 nm wavelengths for excitation and emission, respectively. Trolox was used as standard, and ORAC was expressed as Trolox equivalents in micromole per litre of serum (μmol/L). Final ORAC values were calculated by using a regression equation between the Trolox concentration and the net area under the fluorescein decay curve. Reactions were carried out in triplicate and coefficients of variation were below 10%. As over half of the antioxidant capacity of the human blood is due to uric acid levels, concentrations of uric acid were obtained by enzymatic methods and subtracted from ORAC values before analysis.

Serum HCY levels (μmol/L) were also obtained from each participant using standard enzymatic methods (A15 Random Access Analyzer, Biosystems, Spain).

### PSG recordings

Overnight PSG recordings were performed in a sound-attenuated bedroom with infrared video-controlled supervision. The PSG protocol included 59 EEG standard locations following the International 10–10 System, vertical and horizontal electrooculography, and electromyography of submental muscles. Signals were filtered (0.1–100 Hz bandpass), digitized (250 Hz, 16-bit resolution), and stored for subsequent analyses (BrainAmp MR, Brain Products, Germany). Visual sleep scoring was performed by a trained sleep technician in accordance with standardized criteria^21^, and reviewed by one of the authors of the present study (J.L.C.). Scoring criteria for EEG arousals were taken from the American Sleep Disorders Association report^22^, and the level of sleep fragmentation was determined with the arousal index (AI). This index resulted from dividing the number of arousals in a sleep stage by the time (in hours) spent in that sleep stage.

### Cerebral MRI

Structural cerebral images were acquired on a Philips Achieva 3 T MRI scanner equipped with an 8-channel head coil. One T1-weighted magnetization-prepared rapid gradient echo (MP-RAGE) cerebral image was obtained for each participant. Acquisition parameters were empirically optimized for gray/white contrast (repetition-time = 2300 ms, echo-time = 4.5 ms, flip angle = 8°, matrix dimensions 320 × 320, 0.8 isotropic voxel, no gap between slices, time per acquisition = 9.1 min).

MRI data were preprocessed using the voxel-based morphometry (VBM) approach integrated in SPM12 (Wellcome Trust Center for Neuroimaging; www.fil.ion.ucl.ac.uk/spm). Briefly, T1-weighted brain images were manually reoriented to the anterior commissure and further segmented into different compartments following the unified segmentation approach implemented in SPM12. Next, the diffeomorphic anatomical registration through an exponentiated lie algebra (DARTEL) algorithm was applied to segmented brain images. Gray matter (GM) maps were spatially normalized into the Montreal Neurological Institute (MNI) brain space, and smoothed with an isotropic Gaussian kernel of 12 mm full-width at half-maximum.

### Statistical analysis

Statistical analysis of demographic variables, HCY/TAC levels, and sleep parameters were conducted in SPSS v22 (SPSS inc., USA). Sex differences were inspected using the Chi-square test while the remaining demographic variables were assessed with unpaired t-tests. Group differences in sleep parameters and HCY/TAC levels were evaluated by analyses of covariance (ANCOVA) including age and sex as nuisance when they achieved statistical significance. Linear regression analyses were further conducted in each group to assess if changes in sleep parameters and HCY levels were associated with variations in TAC concentrations, and if HCY levels contributed to explain the variance in objective sleep. These analyses were adjusted by those demographic variables that explained a significant part of the variance in the dependent variable. If regression coefficients achieved statistical significance in at least one group, we proceeded to compare regression coefficients between the two groups.

Based on the general linear model implemented in SPM12, voxel-wise linear regression analyses were performed in each group to assess whether TAC/HCY levels predicted changes in cerebral GM. Again, if the relationship reached significance in at least one group, we proceeded to evaluate group differences in the strength of correlations. These analyses were adjusted by age and sex when they achieved statistical significance. The resulting statistical maps were thresholded at P < 0.05 at the cluster level using the family-wise error (FWE) rate.

**Figure 1:**
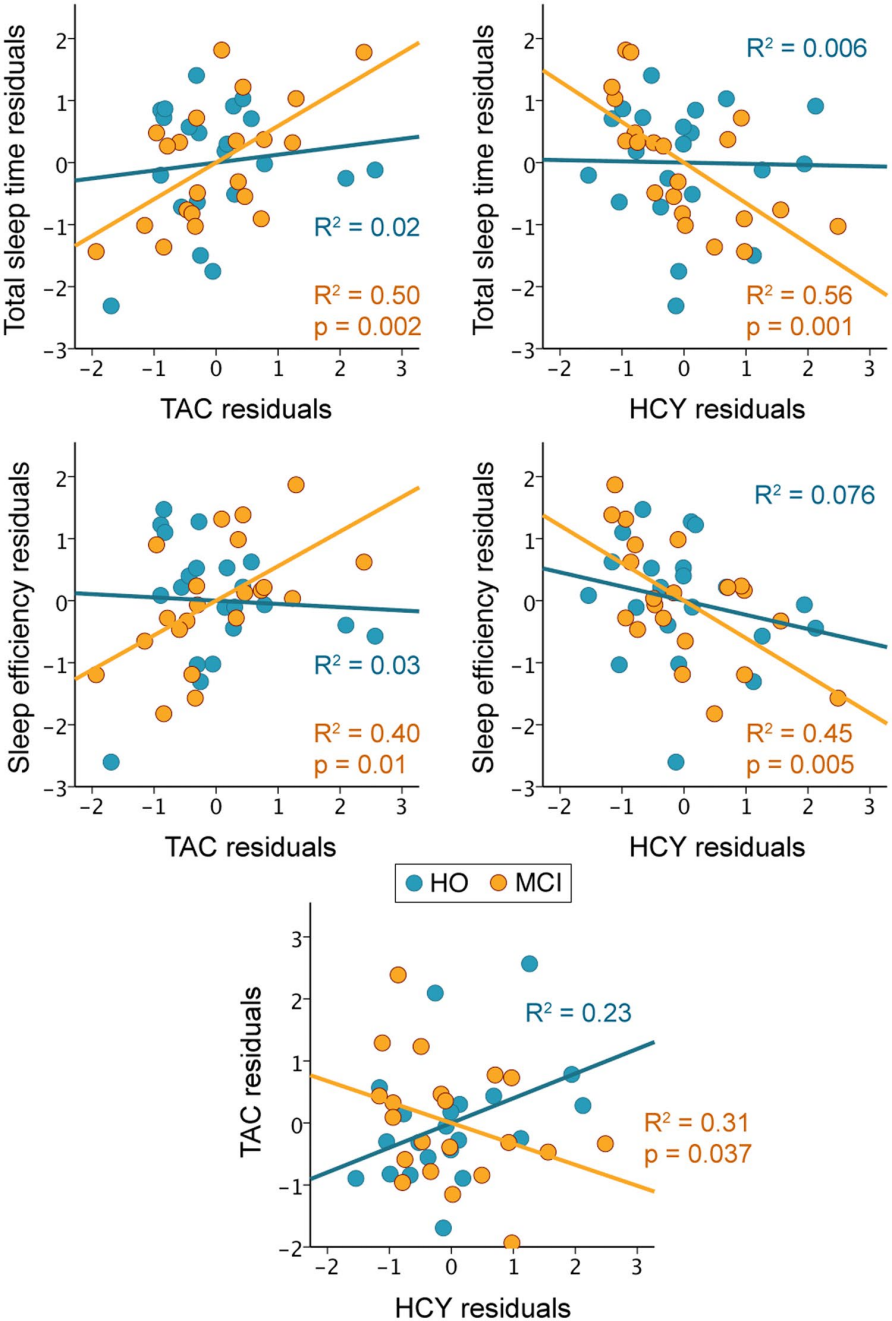
Correlations between TAC, HCY, and sleep. Scatter plots of TAC/HCY against total sleep time/sleep efficiency for HO (in blue) and MCI (in orange). The variables included in the scatter plots correspond to the standardized residuals from a simple regression with age as independent variable. The relationship between HCY and TAC residuals is shown in the bottom panel. The coefficients of determination (R2) and probability (p value) for each group are also indicated.

Path analysis was conducted to examine our initial hypothesis that objective sleep mediates the relationship between HCY and TAC levels in MCI patients. The basic single mediation model is depicted as a path diagram in Fig. 1A,B. Specifically, this model evaluates whether HCY levels explain changes in different sleep parameters (path β1), which in turn are expected to account for variations in TAC (path β2). The path *c*’ represents the relationship between HCY and TAC levels after controlling for the influence of sleep. The single mediators (sleep parameters) were considered to mediate this relationship if the joint significant test (paths β1 and β2) and the product-of-coefficients (β1*β2) were significantly nonzero, and if the total effect of HCY on TAC (path *c*) reached significance. Demographic variables were included as nuisance in the model if they explained a significant part of the variance in the mediator and/or the dependent variable. Inference was determined by 95% bias-corrected bootstrap confidence intervals from 10,000 bootstrap samples. These analyses were performed with the Andrew Hayes’ *PROCESS* macro in SPSS.

If results support the idea that both increased HCY and decreased TAC are associated with GM loss in MCI, we will further test the hypothesis that the relationship between HCY and GM is mediated by the relationship between sleep and TAC. The serial multiple-mediator model used to test this hypothesis is depicted in Fig. 1C.

## Results

### Demographic, cognitive profile, sleep patterns and blood markers

Table 1 shows mean and standard deviations for all variables included in the study for HO and MCI individuals. While both groups were statistically homogeneous in demographics, MCI patients showed lower scores in global cognitive function (P = 0.005), immediate (P = 10^−6^) and delayed memory (P = 10^−8^). As previously reported, REM sleep was significantly shortened (P = 0.03) and slow-wave sleep was more disrupted (P = 10^−5^) in MCI compared to HO subjects^23^. Although both sleep duration and sleep efficiency showed a trend toward reduction in MCI compared to HO, these differences did not achieve statistical significance.

**Table 1:** Demographic, cognitive, sleep, and blood markers.

	HO	MCI
Age, yr	67 ± 5.5	69.8 ± 6.5
Sex (F/M)	10/11	6/15
Education, yr	8.6 ± 4.3	8.2 ± 5.4
CDR (sum of boxes)	0	0.5
MMSE	28.3 ± 1.3	26.7 ± 2.5*
Immediate recall	14.2 ± 2.9	9.3 ± 2.8*
Delayed recall	13.3 ± 2.7	6.5 ± 4*
TST (min)	395.7 ± 30.7	372.6 ± 55.5
Stage 2 (%)	34.6 ± 6.9	36.3 ± 9.3
SWS (%)	23.6 ± 7	22.7 ± 12.2
REM (%)	14.1 ± 3.4	10.1 ± 4.6*
WASO (%)	13.2 ± 5.8	15.6 ± 6.2
AI Stage 2	0.31 ± 0.17	0.25 ± 0.13
AI SWS	0.05 ± 0.03	0.15 ± 0.08*
AI REM	0.18 ± 0.14	0.22 ± 0.09
Sleep efficiency (%)	83.8 ± 6.1	78.6 ± 11.5
TAC (μmol/L)	440.8 ± 301.4	270.5 ± 187.5*
Homocysteine (μmol/L)	14.0 ± 4.9	15.1 ± 4.7

Results are expressed as mean ± standard deviation. CDR: Clinical Dementia Rating; CDR = 0: no dementia; CDR = 0.5: questionable or very mild dementia; MMSE: Mini-Mental State Examination; TST: total sleep time; WASO: wake after sleep onset; SWS: slow-wave sleep; REM: rapid-eye movement sleep; AI: arousal index. *The statistical test for group differences reached significance (*P* < 0.05).

TAC levels, reflected by ORAC values, were significantly lower in MCI compared to HO (P = 0.03). HCY levels did not allow us to distinguish the two groups, although the proportion of MCI patients showing clinical HCY values (>14 μmol/L) was almost twofold greater than in the HO group (47.6% of MCI *vs*. 28.6% of HO). The lack of group differences in HCY levels is likely due to the small sample size employed in the present study.

### Group differences in GM volume

Compared to HO, MCI patients showed a significant reduction of the right hippocampus (P_FWE-corrected_ = 0.002; MNI coordinates of the peak voxel: [33–11–15]), left middle temporal gyrus (P_FWE-corrected_ = 0.001; [−45–36 5]) and right inferior frontal gyrus (P_FWE-corrected_ = 0.001; [32 23 27]).

### Relationship between HCY, TAC and sleep

**Figure 2:**
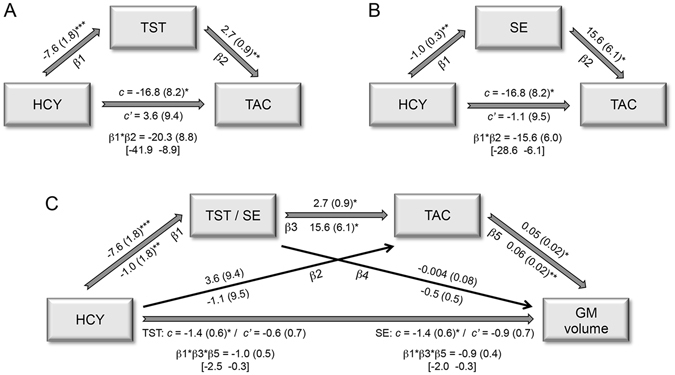
Single and multiple mediation effects. Standardized regression coefficients (standard error) for the relationship between HCY and TAC as mediated by total sleep time (**A**) or sleep efficiency (**B**) in MCI. (**C**) Standardized regression coefficients (standard error) for the relationship between HCY and GM loss as serially mediated by total sleep time/sleep efficiency and TAC in MCI. The standardized regression coefficient (standard error) between HCY and TAC or between HCY and cerebral GM before (c) and after controlling for either sleep or sleep and TAC (c’) are also indicated. Standardized regression coefficients (standard error) for the two-path (β1*β2) and three-path (β1*β3*β5) indirect effects together with the 95% bias-corrected bootstrap confidence intervals from 10,000 bootstrap samples are indicated in the bottom part of each model. *P < 0.05; **P < 0.005; ***P < 0.001.

We examined whether HCY and sleep parameters were associated with TAC levels in each group. Scatter plots showing these results are shown in Fig. 2. Regression analyses revealed that shorter sleep duration (R^2^ = 0.50; F_2,18_ = 8.9; P = 0.002) and lower sleep efficiency (R^2^ = 0.40; F_2,18_ = 6.1; P = 0.01) significantly correlated with decreased TAC levels in MCI patients. No relationship between sleep and TAC levels was found in HO, nor did regression coefficients differ between groups.

Next, we evaluated whether HCY levels predicted changes in objective sleep in each group. Results showed that increased HCY levels were significantly correlated with shorter sleep duration (R^2^ = 0.56; F_2,18_ = 11.3; P = 0.001) and lower sleep efficiency (R^2^ = 0.45; F_2,18_ = 7.4; P = 0.005) in MCI patients. Associations between HCY levels and sleep were absent in HO subjects, and regression coefficients did not significantly differ between groups. However, a stronger association between higher HCY and decreased TAC levels was shown in MCI compared with HO (F_4,37_ = 4.5; P = 0.005; Beta = −1.1; t = −2.3; P = 0.026), although this relationship only reached statistical significance in the MCI group (R^2^ = 0.31; F_2,18_ = 4.0; P = 0.037).

Having characterized the association between HCY, TAC and sleep in MCI patients, we next sought to determine if sleep acted as a mediator of the relationship HCY-TAC. Joint and indirect effects derived from single mediation models indicated that this relationship reached significance in MCI, but not in HO subjects. More specifically, we found that increased levels of HCY resulted in poorer sleep quality (i.e., shorter sleep duration and lower sleep efficiency), which in turn led to significant reductions of TAC levels in MCI patients (Fig. 1A and B, respectively). Conversely, HCY did not mediate the impact of sleep on TAC.

### Serial relationship between HCY, TAC, sleep, and cerebral GM

**Figure 3:**
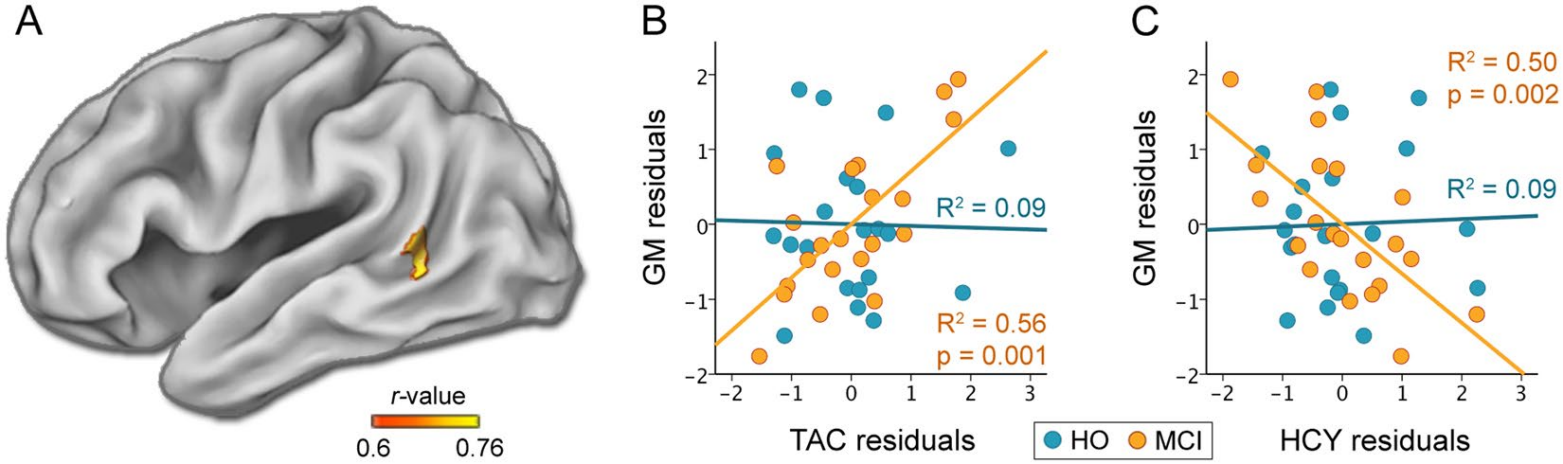
Correlations between HCY/TAC levels and cerebral GM. (**A**) Significant results derived from a linear regression between TAC and GM (p < 0.05; FWE corrected) in MCI. Scatter plots of TAC (**B**) or HCY (**C**) against GM for HO (in blue) and MCI (in orange). The variables included in the scatter plots correspond to the standardized residuals from a simple regression with age as independent variable. The coefficients of determination (R^2^) and probability (p value) for each group are also indicated.

Regression analyses revealed a positive correlation in MCI between TAC and GM in the left middle temporal lobe (P_FWE-corrected_ = 0.002; MNI coordinates of the peak voxel: [−60–57 9]). These results are illustrated in Fig. 3A. Interestingly, GM loss in this region was further associated with increased HCY levels in MCI (P_FWE-corrected_ = 0.014; [−53–54 6]). Group comparison of regression coefficients did not achieve statistical significance. The scatter plots of both analyses are shown in Fig. 3B and C.

Given that GM loss in the left middle temporal lobe was significantly correlated with both increased HCY and decreased TAC levels, we tested the serial multiple-mediation effect illustrated in Fig. 1C. Results derived from this model showed that joint effects and the three-path indirect effect reached statistical significance for both total sleep time and sleep efficiency, but only in MCI. Reversing the direction of the mediation (HCY → TAC → sleep → GM loss) and of the dependent and independent variables (GM loss → sleep → TAC → HCY) yielded non-significant results.

## Discussion

The present study provides the first evidence that poorer sleep quality in MCI patients, in addition to being significantly associated with reduced TAC and increased HCY levels, mediated the relationship between HCY and TAC, two factors actively involved in the pathological cascade of AD^4, 10^. Furthermore, we found that HCY-related cerebral GM loss in MCI was determined by the serial relationship between poorer sleep quality and decreased TAC levels, revealing a complex association between risk factors and sleep from prodromal AD stages. While still correlative in nature, these findings lends credence to the hypothesis that HCY activates a damage process that disturbs methylation and/or redox homeostasis by enhancing Aβ neurotoxicity^24^, and that poorer sleep quality may play a key role in exacerbating this pernicious cycle in MCI patients. Given the modifiable nature of these factors, our results raise the possibility that tailoring interventions aimed at both lowering HCY levels and improving sleep quality may contribute to slowing cognitive decline and disease progression in preclinical and prodromal AD stages.

**Figure 4:**
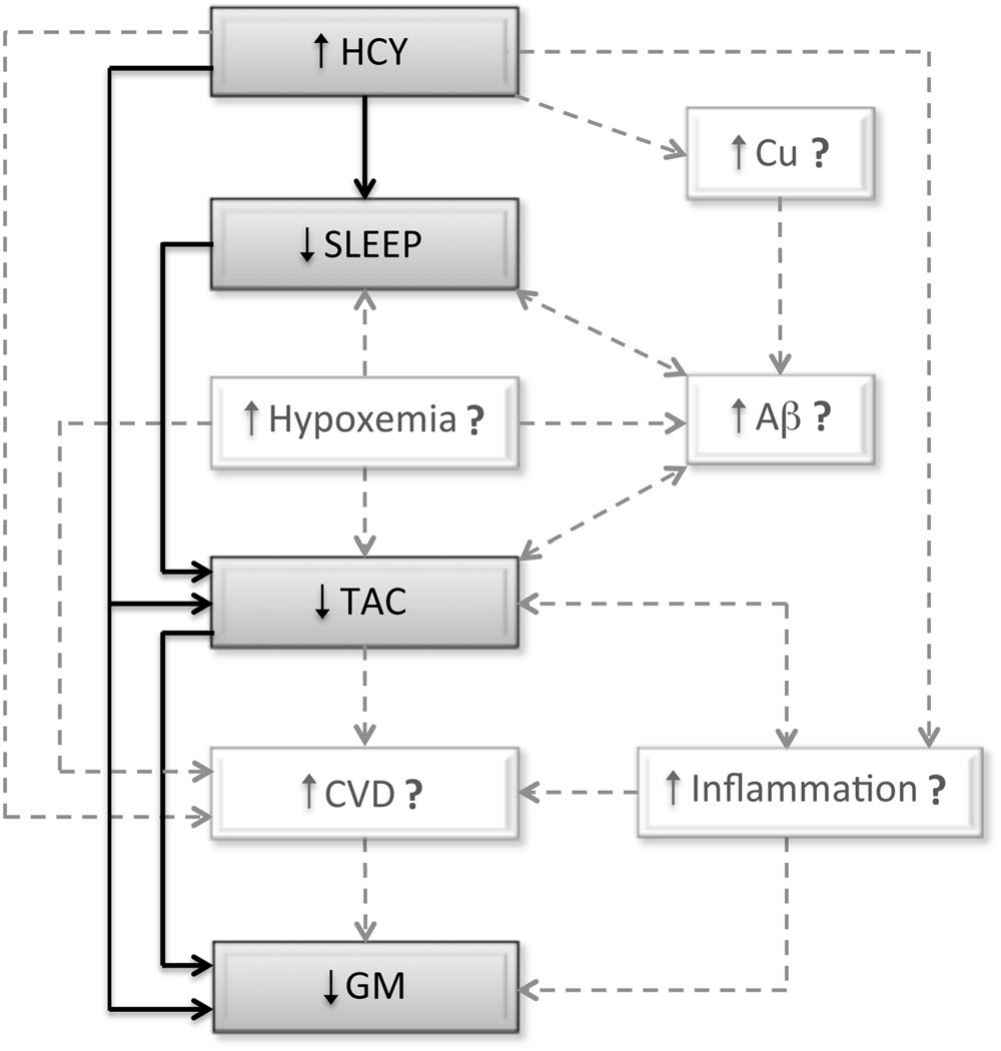
Hypothetical model linking our results with other potentially intervening factors. Gray boxes and black solid arrows are based on findings obtained in the current study; while white boxes and gray dashed arrows refer to other potentially intervening elements derived from alternative evidence. Our findings suggest that the relationship between HCY and TAC levels was mediated by poorer sleep quality in MCI, while the impact of HCY on cerebral GM was mediated by the relationship between sleep quality and TAC levels. Based on previous evidence, we hypothesize that the impact of HCY on TAC levels may be further mediated by copper (Cu) accumulation and/or Aβ aggregation in the brain, and that sleep disruptions may be additionally prompted by increased hypoxemia, which in turn has also shown to enhance Aβ burden; whereas the impact of HCY on GM loss may be further mediated by cardiovascular dysfunctions (CVD), which is promoted by multiple factors including elevated HCY, oxidative stress, reduced TAC and low-grade systemic inflammation.

To the best of our knowledge, this is the first study to pinpoint an association between objective sleep and TAC in prodromal AD. Our results reveal that both shorter sleep duration and lower sleep efficiency were correlated with decreased TAC in MCI patients. We postulate that increased levels of soluble Aβ species and/or Aβ aggregates may be a key factor in determining such an association, promoting a vicious cycle wherein poor sleep and Aβ accumulation are mutually strengthened, subsequently producing oxidative damage that leads to increased Aβ burden, which in turn contributes to the reinforcement of this positive-feedback loop. This hypothetical model, illustrated in Fig. 4, is based on evidence supporting the existence of a bi-directional relationship between sleep and Aβ deposition^25^, and between Aβ aggregation and Aβ-induced oxidative stress^26^. Regarding the sleep-Aβ relationship, previous studies have shown that sleep loss increases CSF Aβ levels in rodents^14^ and humans with presenilin mutations^27^. Further, the sleep-wake cycle is significantly altered following plaque formation in transgenic mouse models of amyloidosis^27^. In the relationship between Aβ and oxidative stress, evidence suggests that Aβ accumulation induces ROS that in turn increases Aβ production and aggregation, ultimately accelerating the progression of AD^28^. This model is complemented by studies that demonstrate a relationship between sleep loss and increased oxidative stress with age^12^.

The relationship between poorer sleep quality and reduced TAC levels in MCI could also be due to the presence of subclinical sleep-disordered breathing, characterized by recurrent arousals from sleep and intermittent hypoxemia. In fact, sleep-disordered breathing has been further associated with advanced cognitive decline in the elderly^29^ and greater Aβ burden in MCI^30^. In the light of these considerations, previous studies have shown that hypoxia causes sleep fragmentation, both conditions related to increased inflammation and aggravation of AD pathology^31^. Moreover, hypoxia produces oxidative damage to different cellular compartments, especially in the vascular endothelium and neuronal cell bodies^32^, and increases cellular apoptosis in hippocampus and cortex^33^. Unfortunately, we were unable to rule out the presence of sleep-disordered breathing disorders in our study, since respiratory measures were not included in the PSG montage. Future studies should therefore clarify whether sleep-disordered breathing contributes independently or synergistically to the relationship between impaired sleep and lower TAC levels in prodromal AD.

Our findings provided the first evidence of correlations between increased HCY levels and poorer sleep quality in MCI patients. Previous studies have revealed that combined exposure to HCY and copper promotes oxidative damage and enhances Aβ neurotoxicity in neuronal cultures^34^. These findings are complemented by others showing that copper is associated with increased CSF Aβ levels in AD patients^35^, likely because copper accumulation in brain vessels disrupts Aβ clearance^36^. Whether the serial relationship between HCY, copper and Aβ aggregation is affecting sleep in MCI/AD is a testable hypothesis for future experiments. On the other hand, elevated concentrations of HCY have been regarded as an independent risk factor for cardiovascular disease^37^, which in turn is associated with sleep-related breathing disorders^38^ and has shown to substantially increase dementia risk^39^. Therefore, the relationship between HCY levels and poorer sleep found in MCI patients may be due to a variety of indirect factors associated with sleep fragmentation, all of them instrumental in the development of dementia. These hypotheses are illustrated in Fig. 4.

We further demonstrated that decreased TAC and increased HCY levels in MCI were correlated with GM loss in regions of the middle temporal lobe. While our findings provide the first evidence of TAC-related GM loss in prodromal AD, HCY-related structural brain changes had been previously reported^11^. Here we have reached another milestone by showing that the impact of increased HCY levels on GM loss may further depends on the serial relationship between sleep and TAC in MCI patients. At the cerebral level, Aβ burden, resulting from increased HCY, poor sleep, and oxidative damage may exacerbate this complex relationship in MCI. However, other aging-related peripheral factors associated with HCY and oxidative stress, such as cardiovascular dysfunction and systemic inflammation, may also mediate or modulate the influence on GM loss (Fig. 4). Aging is associated with a chronic low-grade proinflammatory state characterized by an elevation in circulating levels of cytokines and acute phase proteins in the absence of overt infection^40, 41^. This phenomenon, often referred to as “inflammaging”^42^ and promoted by a wide range of factors including oxidation, age-related changes in fat tissue redistribution and sleep loss, and chronic disorders such as cardiovascular disease and AD, has been shown to have consequences for cognitive health in both normal and pathological aging^43^. Although the neural bases accounting for the link between inflammation and cognitive decline are far to be understood, recent evidence suggests that cerebral GM loss may mediate this relationship^44^. Inflammation, which is reciprocally related to oxidative stress^45^, not only makes elderly persons more susceptible to cardiovascular disease, but may also act as a determining factor in the relationship between HCY and cardiovascular dysfunction^46^, a condition that has also been associated with detrimental effects on cerebral GM^47^.

Although preliminary due to the small sample size, our findings offer novel empirical foundations to modify lifestyle risk factors aimed at promoting cognitive health in the elderly. For instance, weaken the association between HCY and TAC levels by enhancing sleep quality may be beneficial in slowing cognitive decline in older adults who are at risk of developing AD. Recent evidence suggests that poor sleep quality was correlated with cognitive decline in normal elderly subjects after 3 years of follow-up^48^. Consequently, the identification of impaired sleep in middle age may offer opportunities to prevent cognitive decline late in life. Furthermore, HCY is a risk factor for brain atrophy^11^ and could cause cognitive impairment via direct neurotoxicity or by reinforcing other biological pathways including those of Aβ^49^. Previous studies have shown that treatment with B vitamins in normal elderly subjects with elevated HCY reduces the rate of brain shrinkage and delays cognitive decline, these interventions being more beneficial in those individuals whose patterns of brain atrophy has not yet reached critical levels^50^. Therefore, improving B-vitamin status in asymptomatic elderly subjects may play an important role in preventing age-related cognitive impairment and decline^49^. Folic acid supplementation could also be equally advantageous for cognitive function in MCI by lowering peripheral levels of inflammatory cytokines^51^ and diminishing GM loss in cerebral regions vulnerable to AD^52^. Finally, resistance training, especially in overweight and obese older adults, may also provide some protection against cardiovascular risk factors by lowering exercise-induced oxidative stress and HCY^53^.
